# Preliminary Evidence on the Efficacy and Tolerability of Quetiapine in Dual Disorders: A Prospective, Multicentric, Real-World Study

**DOI:** 10.3390/ph19030423

**Published:** 2026-03-05

**Authors:** Alessio Mosca, Clara Cavallotto, Stefania Chiappini, Giacomo d’Andrea, Francesco Di Carlo, Carlotta Marrangone, Rita Allegretti, Nicola Ciraselli, Maria Pepe, Luigi Dattoli, Beatrice Petrosino, Andrea Di Cesare, Valerio Ricci, Marco Di Nicola, Mauro Pettorruso, Giovanni Martinotti

**Affiliations:** 1 Department of Neurosciences, Imaging and Clinical Sciences, “G. D’Annunzio” University, 66100 Chieti, Italy; alessio.mosca909@gmail.com (A.M.); giacomo.dandrea1993@gmail.com (G.d.); francesco.dic@hotmail.it (F.D.C.); carlotta.marrangone@gmail.com (C.M.); rita.alle@gmail.com (R.A.); n.ciraselli@gmail.com (N.C.); luigidattoli4337@gmail.com (L.D.); mauro.pettorruso@hotmail.it (M.P.); giovanni.martinotti@gmail.com (G.M.); 2 School of Medicine, UniCamillus International Medical School University, Via di S. Alessandro 8, 00131 Rome, Italy; stefaniachiappini9@gmail.com; 3 University Policlinic Foundation “A. Gemelli” IRCSS-Catholic University of the Sacred Heart, Largo Agostino Gemelli, 8, 00136 Rome, Italy; mariapepe.992@gmail.com (M.P.); marcodinicola.md@gmail.com (M.D.N.); 4 SerD Caltagirone, UOC Dipendenze Patologiche, ASP3 Catania, 95100 Catania, Italy; beatricepetrosino.bp@gmail.com; 5 SerD Via dei Riari, ASL Roma1, 00131 Rome, Italy; andreadc25@gmail.com; 6 Department of Psychiatry, “San Luigi Gonzaga” Hospital, University of Turin, 10124 Turin, Italy; v.ricci@sanluigi.piemonte.it

**Keywords:** dual disorders, substance use disorder, quetiapine, real-world study, addiction, substance-induced psychosis, atypical antipsychotic, schizophrenia spectrum disorder

## Abstract

**Background**: Dual disorders (DDs) describe the coexistence of substance use disorder (SUD) and another mental health condition, commonly within psychotic and affective categories. These conditions represent a significant challenge in clinical management due to their bidirectional interactions and complexity. This study aims to evaluate the efficacy and safety of quetiapine, a second-generation antipsychotic, in patients with schizophrenia spectrum disorders and comorbid substance use disorders. **Methods**: A total of 28 participants with schizophrenia spectrum disorder and comorbid SUD underwent psychometric evaluations at baseline (T0), one month (T1) and three months post-initiation of quetiapine treatment (T2), administered at a mean dosage of 165 mg/day. Key outcome measures included psychopathological burden (PANSS), aggressivity (MOAS), substance craving (VAS Craving), and quality of life (Q-LES-Q-SF scales). **Results**: Quetiapine demonstrated significant reductions in psychopathological symptoms, with decreased PANSS total scores (*p* < 0.001). Positive symptoms (*p* < 0.001), negative symptoms (*p* = 0.002), substance craving (*p* = 0.001), and aggressivity (*p* = 0.006) also showed notable reductions. Quality of life significantly improved across Q-LES-Q-SF scores (*p* < 0.001). Quetiapine was well-tolerated, with no dropouts related to drug-induced side effects. **Conclusions**: This study provides preliminary evidence supporting the efficacy and safety of quetiapine in individuals with dual disorders. Improvements in psychopathology, substance craving, and quality of life underscore the importance of integrating tailored and comprehensive treatment strategies to address the multifaceted challenges of this challenging population.

## 1. Introduction

Dual disorders (DDs) denote the presence of at least one substance use disorder (SUD) in association with another mental disorder, most frequently within the psychotic or affective spectrum. Initially identified in the 1980s and increasingly acknowledged during the 1990s, DDs have been conceptualized through various definitions, and a universally agreed-upon framework has yet to be established. The World Health Organization (WHO) originally defined DDs as “the co-occurrence in the same individual of a psychoactive substance use disorder and another psychiatric disorder” [1]; however, contemporary perspectives have broadened this concept to include both concurrent and sequential manifestations of substance use and other mental disorders within the same individual across the lifespan [2]. This definition underscores the complexity and overlapping nature of these conditions, which present significant challenges for healthcare providers.

The intricate interplay between psychoactive substances and psychiatric disorders is characterized by bidirectional causality, wherein substance misuse may precipitate the onset of mental illness, and mental illness can, in turn, lead to substance misuse. This mutual exacerbation compounds the severity and progression of both conditions [3]. The clinical picture is further complicated by the heterogeneity of substance-related effects and the broad spectrum of psychiatric disorders involved, each contributing distinct clinical trajectories and behavioral challenges. Psychotic symptoms associated with the use of cannabis and novel psychoactive substances (NPSs) [4] exemplify the difficulty in differentiating *substance-induced psychosis*, a formal diagnostic entity, from primary psychotic disorders [5,6,7]. In addition, psychotic manifestations may also arise in the context of *drug-induced psychosis*, a broader descriptive term often used to indicate psychotic symptoms secondary to specific substances or medications [8]. In both substance-induced and drug-induced psychotic conditions, the prevalence of dual disorders is markedly higher than in the general population, as confirmed by several meta-analyses documenting the substantial comorbidity between substance use disorders and other psychiatric conditions [9]. For example, approximately one-fifth of individuals with eating disorders develop a SUD at some point in their lives [10], while around 40% of individuals with schizophrenia spectrum disorders are estimated to experience a comorbid SUD over the lifetime, according to a large systematic review and meta-analysis [11]. Similarly, cannabis use disorder (CUD) is particularly prevalent among individuals with bipolar disorder [9]. These comorbidities are associated with profound social and psychopathological difficulties, including reduced quality of life, encounters with law enforcement, and homelessness [12]. Among individuals with SUD, a dual diagnosis often correlates with exposure to childhood trauma, multiple SUD diagnoses, suicide attempts, and diminished social support [9].

Despite these challenges, authoritative diagnostic manuals such as the DSM-5 do not provide specific criteria for dual disorders, complicating accurate diagnosis and determination of prevalence [13]. Nevertheless, the rising prevalence of dual disorders highlights the need for effective clinical management strategies. The use of atypical antipsychotics, which can target not only psychotic symptoms but also affective and negative symptom dimensions, represents a promising therapeutic approach in these complex cases [1,14]. This is particularly relevant in schizophrenia, where affective and negative symptoms may partially overlap at the clinical level or present independently, yet differ in their underlying mechanisms, longitudinal course, and responsiveness to treatment. While affective symptoms, such as depressive and anxiety features related to mood dysregulation, tend to be more fluctuating and potentially treatment-responsive, negative symptoms reflect more enduring deficits in motivation, emotional expression, and social functioning. Although the available evidence remains limited, recent studies have reported encouraging results with antipsychotics characterized by complex dopaminergic and serotonergic modulation, suggesting a potential role for this pharmacological strategy in improving both psychiatric symptoms and substance-related behaviors.

Real-world investigations examining the use of antipsychotic medications in patients with comorbid substance use disorders remain relatively limited, although they are steadily increasing. Early evidence suggests that several atypical antipsychotics, including clozapine, risperidone, and olanzapine, may exert beneficial effects not only on psychotic symptomatology but also on substance use patterns in individuals with dual disorders [15,16,17,18,19,20,21]. These clinical observations have been largely attributed to shared pharmacodynamic properties, particularly the combined modulation of dopaminergic and serotonergic systems, which play a central role in both psychosis and addictive behaviors.

Within this pharmacological framework, growing attention has been directed toward second-generation antipsychotics characterized by broader and more balanced receptor-binding profiles. Lurasidone, for example, exerts its therapeutic effects through dopamine D_2_ and serotonin 5-HT_2_A antagonism, together with high affinity for 5-HT_7_ receptors and partial agonism at 5-HT_1_A receptors [22,23]. This receptor configuration has been associated with improvements in psychotic symptoms, affective regulation, and cognitive functioning, while maintaining a relatively favorable metabolic profile [23].

Similarly, brexpiprazole presents a pharmacodynamic profile that overlaps substantially with that of quetiapine and other atypical antipsychotics, acting as a partial agonist at D_2_/D_3_ and 5-HT_1_A receptors and as an antagonist at 5-HT_2_A receptors. Such balanced dopaminergic–serotonergic modulation has been linked to clinical benefits across both psychiatric symptom domains and substance-related outcomes in patients with dual diagnosis [24,25,26].

Taken together, these findings support the concept of a pharmacological continuum among atypical antipsychotics, in which shared receptor affinities, particularly involving D_2_, 5-HT_2_A, 5-HT_1_A, and 5-HT_7_ receptors, may underlie their therapeutic potential in complex and comorbid psychiatric conditions. Within this continuum, quetiapine emerges as a particularly relevant candidate for further investigation.

Quetiapine is an atypical antipsychotic approved by the U.S. Food and Drug Administration (FDA) for the treatment of schizophrenia, acute manic episodes, and as adjunctive therapy for major depressive disorder. It is available in both immediate-release and extended-release formulations, with recommended adult dosages ranging from 150 to 750 mg/day [27]. Pharmacodynamically, quetiapine shares multiple receptor-level mechanisms with lurasidone and brexpiprazole, including antagonism at 5-HT_2_ receptors and modulation of dopaminergic D_1_ and D_2_ receptors [28]. Through its actions within mesocortical and mesolimbic pathways, quetiapine contributes to the regulation of dopaminergic dysregulation implicated in both positive and negative symptoms of schizophrenia.

Beyond its antipsychotic properties, quetiapine has also been explored for its potential effects on substance use and cravings in patients with comorbid schizophrenia-spectrum and substance use disorders. An open-label trial reported reductions in substance consumption and cravings alongside improvements in psychiatric symptoms, supporting the hypothesis that quetiapine may exert a dual therapeutic effect on psychotic and addictive dimensions [29].

Furthermore, the anxiolytic and antidepressant properties of quetiapine and its active metabolite norquetiapine are thought to be mediated by inhibition of the norepinephrine transporter (NET) and partial agonism at 5-HT_1_A receptors, respectively [30]. Additional evidence suggests that antagonism at 5-HT_2_A and 5-HT_7_ receptors may further contribute to its antidepressant efficacy, reinforcing its pharmacological continuity with other second-generation antipsychotics [27].

Given the pharmacological properties of quetiapine and its broad therapeutic profile, the objective of this study was to evaluate its efficacy and safety in patients with schizophrenia spectrum disorders, comorbid with substance use disorders, as a clinical population characterized by prominent psychotic and affective dysregulation and limited evidence-based treatment options. We hypothesized that quetiapine may lead to improvements not only in psychotic symptoms and affective instability, but also in substance use-related outcomes, including cravings, while demonstrating an acceptable safety and tolerability profile in this clinically complex population.

## 2. Results

### 2.1. Baseline Characteristics

A total of 28 subjects were enrolled in the study (M: 22/F: 6; mean age: 31.4 ± 13.4 years), and a comprehensive presentation of their sociodemographic and clinical information is detailed in Table 1. Regarding psychiatric diagnoses, the majority presented with substance-induced psychosis (*n* = 15; 53.6%), followed by schizoaffective disorder (*n* = 7; 25%) and unspecified psychosis (*n* = 6; 21.4%). Concerning SUD, cannabis was the most commonly reported substance (*n* = 12; 42.9%), followed by cocaine (*n* = 5; 17.9%) and alcohol (*n* = 3; 10.7%). Significantly, a substantial proportion of the participants were characterized as polysubstance users (*n* = 8; 28.5%), emphasizing the gravity of the situations of the patient population investigated in this study.

Regarding pharmacological treatment, at baseline, most subjects (*n* = 22; 78.6%) were antipsychotic and drug-free, so they were immediately initiated on quetiapine. Conversely, six patients (21.4%) were already receiving ongoing antipsychotic treatment at baseline and were subsequently transitioned to quetiapine through a cross-tapering procedure. Prior antipsychotic therapies included aripiprazole (*n* = 1), paliperidone (*n* = 1), haloperidol (*n* = 1), olanzapine (*n* = 1), clotiapine (*n* = 1), and risperidone (*n* = 1). In terms of other medications prescribed apart from quetiapine, 10 patients (35.7%) were taking mood stabilizers, 9 (32.1%) were on antidepressants, and 12 (42.9%) were prescribed anxiolytics. A minority of patients (*n* = 3; 10.7%) were undergoing substitution therapy, while 2 (7.1%) received other treatments. Finally, thirteen patients (46.4%) were concurrently engaged in structured psychotherapeutic interventions.

### 2.2. Efficacy and Safety

**Figure 1:**
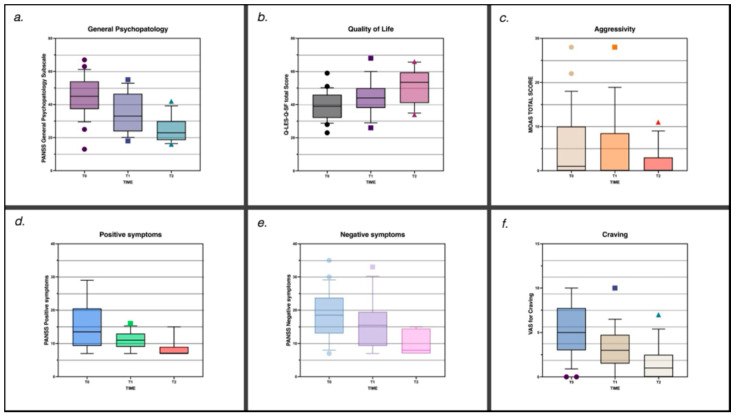
Temporal trends in psychopathology, functioning, aggression, and craving across the 3-month observation period. Box-and-whisker plots depict distributions at baseline (T0), 1-month follow-up (T1), and 3-month follow-up (T2) for (**a**) PANSS General Psychopathology; (**b**) Quality of Life (Q-LES-Q-SF total score); (**c**) MOAS; (**d**) PANSS Positive Symptoms; (**e**) PANSS Negative Symptoms; and (**f**) VAS for Craving.

Three months of treatment with quetiapine, at an average dosage of 164.3 ± 146.9 mg (25–400 mg/day) once daily, proved effective in reducing the overall psychopathological burden, as indicated by significant improvements in *PANSS* scores. The mean score on the *positive symptoms subscale* decreased from T0 = 15.4 ± 6.9 (95% CI: 11.63–19.14) to T1 = 11.3 ± 3.1 and T2 = 8.7 ± 2.9 (95% CI: 7.12–10.26) (**F(2,24) = 12.846**, *p* < 0.001, **η^2^ₚ = 0.517**); the *negative symptoms subscale* decreased from T0 = 16.2 ± 7.0 (95% CI: 12.43–20.03) to T1 = 12.77 ± 4.5 and T2 = 9.8 ± 3.5 (95% CI: 7.93–11.76) (**F(2,24) = 7.933**, *p* < 0.001, **η^2^ₚ = 0.398**); and the *general psychopathology subscale* decreased from T0 = 40.5 ± 13.3 (95% CI: 33.25–47.67) to T1 = 32.7 ± 11.18 and T2 = 24.6 ± 7.7 (95% CI: 20.45–28.78) (**F(2,24) = 10.560**, *p* < 0.001, **η^2^ₚ = 0.468**). Similarly, *MOAS* scores showed a significant reduction in aggressivity from T0 = 6.9 ± 6.7 (95% CI: 3.27–10.57) to T1 = 4.0 ± 5.5 and T2 = 1.8 ± 3.4 (95% CI: 0.00–3.69) (**F(2,24) = 6.117**, *p* = 0.007, **η^2^ₚ = 0.338**), and the *VAS* score decreased from 4.9 ± 2.3 (95% CI: 3.66–6.19) at baseline to 2.69 ± 1.7 at T1 and 1.6 ± 2.0 at T2 (95% CI: 0.54–2.69) (**F(2,24) = 8.430**, *p* = 0.002, **η^2^ₚ = 0.413**), highlighting the treatment’s impact on reducing substance craving. Quality of life also improved substantially, with the mean *Q-LES-Q-SF* score rising from T0 = 39.6 ± 9.4 (95% CI: 34.06–45.21) to T1 = 45.9 ± 9.77 and T2 = 51.5 ± 11.1 (95% CI: 44.89–58.02) (**F(2,20) = 16.152**, *p* < 0.001, **η^2^ₚ = 0.618**), representing a clinically relevant enhancement (Table 2; Figure 1).

**Figure 2:**
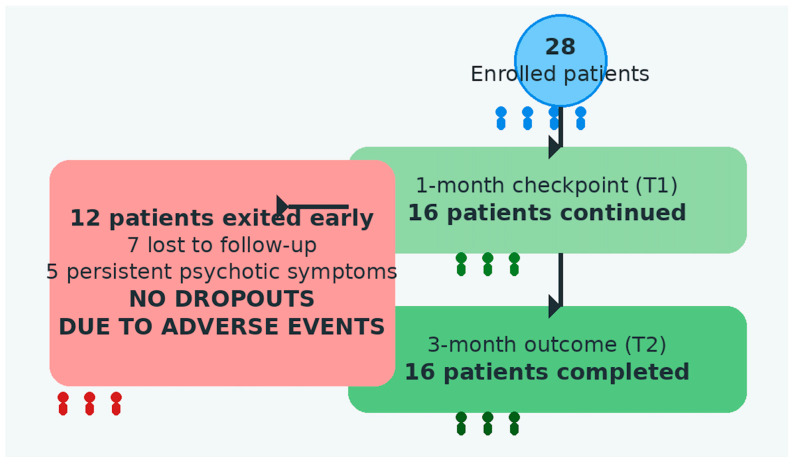
Study flow diagram and patient disposition across follow-up assessments. A total of 28 patients were enrolled at baseline. At the 1-month follow-up (T1), 16 patients continued treatment and clinical monitoring. Twelve patients exited the study prematurely, including 7 patients lost to follow-up and 5 patients due to persistent psychotic symptoms requiring alternative clinical management. No dropouts were attributable to treatment-emergent adverse events.

Finally, dropout rates indicated that 12 patients (42.8%) discontinued treatment before T2. Among these, 7 subjects (25%) were lost to follow-up, and 5 patients (17.9%) were discontinued due to persistence of psychotic symptoms, as shown in Figure 2. Importantly, no dropouts were related to drug-induced side effects. 

## 3. Discussion

This study provides an in-depth evaluation of the use of quetiapine, an atypical antipsychotic, in managing patients with schizophrenia spectrum disorders, comorbid with substance use disorders. This dual diagnosis population often presents unique challenges, with both psychotic symptoms and substance use intricately influencing the course of illness and treatment outcomes.

In the final analysis, our study included 28 participants, the majority of whom were young males, a demographic pattern consistent with the epidemiological profile typically observed in dual-diagnosis populations [11,31,32]. The most prevalent substance use disorder within the sample was related to cannabis, followed by cocaine and alcohol. Notably, a substantial proportion of participants (28.5%) met criteria for polysubstance use, reflecting the clinical complexity and severity commonly reported in this patient group and aligning with findings from previous studies [33,34]. In line with this substance use profile and in accordance with the DSM-5 classification of schizophrenia spectrum disorders, more than half of the participants (*n* = 15) were diagnosed with substance-induced psychosis, followed by schizoaffective disorder (*n* = 7) and unspecified psychotic disorder (*n* = 6). This diagnostic distribution reflects the marked clinical heterogeneity of the sample and underscores the complexity of real-world psychotic populations. Consequently, the generalizability of the findings should be interpreted with caution.

Quetiapine’s pharmacological profile has shown efficacy in addressing psychotic symptoms, both positive and negative, while also alleviating mood-related symptoms. Importantly, substance craving showed a marked decline according to VAS assessments, which was paralleled by a reduction in aggressiveness as measured with the MOAS. These findings suggest a general mood-stabilizing effect of quetiapine, accompanied by an enhancement in overall quality of life, observed in Q-LES-Q-SF scores. This enhancement, likely driven by the reduction in psychopathological burden, further supports its potential as a valuable therapeutic option for this clinically complex population [1,14].

Furthermore, quetiapine’s potential to reduce substance cravings aligns with preclinical findings, suggesting that its dopaminergic modulation may influence drug-seeking behaviors [35,36]. Quetiapine has also demonstrated efficacy in addressing cognitive dysfunctions such as disorganized thinking and memory deficits, attributed to its serotonin receptor activity, which may improve cognitive control over craving [37,38].

Moreover, the positive effects of quetiapine in reducing substance craving may be understood in light of the self-medication hypothesis, which posits that substance abuse represents a strategy for managing anxiety and/or depressive states. From this perspective, it is important to highlight that quetiapine, through its binding to histamine H1 receptors, exerts a sedative effect that may help reduce anxiety and improve sleep quality, often impaired in individuals with substance use disorders [39].

It is also well known that substances exert their addictive potential via activation of the limbic reward system, a process mediated by dopamine release [40]. Drugs acting through dopamine blockade may interfere with this mechanism but can also trigger a compensatory effect, potentially leading to increased substance consumption. This dynamic may explain the rise in substance use observed following treatment with typical antipsychotics, which are known to strongly block dopamine D2 receptors. In contrast, atypical antipsychotics such as quetiapine are characterized by a much weaker dopamine blockade, which is less likely to be associated with increased drug consumption. Specifically, quetiapine produces only transient dopamine receptor blockade within the limbic system, thereby minimizing interference with reward pathways [41].

In addition, other mechanisms may contribute to quetiapine’s ability to reduce substance use, such as modulation of serotonergic neurotransmission, which may play a significant role [42]. In this regard, it is well established that quetiapine exerts a mood-level modulatory effect on serotonergic neurotransmission, a property that has led to its approval, both in Italy and the United States, as a first-line treatment, even in monotherapy, for bipolar depression [43]. This feature may further explain its greater efficacy observed in our sample, composed predominantly of patients presenting with a predominance of negative symptoms.

In this context, it is important to interpret our findings in light of the growing body of evidence on other antipsychotics used in dual-diagnosis populations, particularly second-generation and atypical agents recently evaluated in real-world clinical settings, such as lurasidone [22] and Brexpiprazole [25,26]. Real-world studies investigating lurasidone in patients with schizophrenia spectrum disorders and comorbid alcohol or substance use disorders have reported significant reductions in positive symptoms, craving, and aggressive behaviors, together with improvements in quality of life and overall functioning. These benefits have been observed alongside a favorable safety and tolerability profile, with a low incidence of metabolic adverse effects. Notably, lurasidone’s receptor-binding profile—characterized by high affinity for 5-HT_7_ receptors and partial agonism at 5-HT_1_A receptors—may confer specific advantages in modulating mood, cognition, and emotional regulation, domains that are frequently compromised in individuals with dual disorders [22]. These observations partially align with our results, supporting the hypothesis that antipsychotics with prominent serotonergic modulation may play a meaningful role in reducing craving and improving global clinical outcomes in this population. Similarly, brexpiprazole, a dopamine D_2_ partial agonist with significant activity at 5-HT_1_A and α_1_-adrenergic receptors, has shown promising effects in patients with substance use disorders and comorbid psychiatric symptoms. Available evidence highlights improvements in craving, anxiety, and overall clinical stability, coupled with excellent tolerability, which has been attributed to its balanced dopaminergic modulation and low risk of extrapyramidal side effects [25,26].

Within the same pharmacological framework, evidence has also emerged for aripiprazole in the treatment of dual-diagnosis patients. Observational data on once-monthly long-acting injectable aripiprazole have shown clinical stabilization and improvements in substance use patterns among individuals with schizophrenia and comorbid substance use disorders [44]. In addition, a randomized controlled trial in patients with schizophrenia and cocaine dependence reported a delayed but significant reduction in cocaine craving compared with perphenazine, suggesting a potential modulatory effect on reward-related mechanisms [45].

Regarding safety and tolerability, quetiapine was generally found to be safe and well-tolerated, with no dropouts related to drug-induced side effects. However, a high attrition rate was observed, with only 16 participants completing the 3-month follow-up assessment. This substantial dropout, which included both loss to follow-up (*n* = 7) and treatment discontinuation due to insufficient clinical response (*n* = 5), appears closely related to the intrinsic clinical characteristics of the study population, known to present significant instability and difficulties in treatment adherence. Such findings are consistent with previous evidence reported in the literature, including data from systematic reviews and clinical trials, highlighting the persistent challenges of maintaining longitudinal engagement in this patient group [46,47,48].

Such findings suggest a potential role for integrated treatment approaches targeting both psychotic symptoms and substance use, although further controlled studies are needed to confirm these observations and inform clinical decision-making. Collaboration between mental health and addiction specialists is crucial, alongside regular monitoring and patient education on medication adherence and relapse prevention. Ultimately, the findings underscore the importance of expanding community-based psychosocial interventions, transitioning care beyond residential facilities to ensure sustained patient engagement and support. By addressing the medical, social, and psychological dimensions of dual diagnosis, such an integrated framework can enhance treatment outcomes and improve the quality of life for affected individuals.

### Strengths and Limitations of the Study

Despite the important strength represented by its multicentric design, involving multiple hospitals across different Italian regions, the present study also has several limitations. First, the non-randomized study design, while reflecting real-world clinical practice, represents a limitation. Second, the relatively limited sample size underscores the necessity for larger-scale investigations to strengthen the reliability and generalizability of the findings. Moreover, the absence of a comparison group, such as patients receiving alternative antipsychotics or a placebo, substantially narrows the interpretability of the findings and precludes any direct conclusions regarding the relative efficacy of quetiapine. Furthermore, the relatively short duration of follow-up represents an additional limitation, underscoring the need for longer observational periods to better evaluate the long-term safety profile and sustained therapeutic benefits of quetiapine in this complex population. In this regard, an additional limitation is the lack of a standardized approach for evaluating the adverse events of quetiapine. At present, longitudinal data on its sustained efficacy and tolerability remain limited, warranting cautious interpretation, particularly in specific clinical subpopulations. Another limitation concerns the absence of a standardized and objective approach to evaluating treatment adherence. Owing to the exploratory real-world design and the small sample size, multivariate analyses could not be reliably performed. Therefore, two-tailed statistical tests were employed as a conservative analytical approach appropriate for an exploratory, real-world clinical study. Considering the small sample size and the absence of well-established treatment guidelines, this strategy allowed for the detection of effects in both directions while minimizing the risk of Type I error. In addition, the clinical heterogeneity of the sample, although representative of real-world psychiatric practice, may have introduced variability in treatment response. Accordingly, the results should be interpreted within this methodological framework.

Furthermore, given the real-world and uncontrolled nature of this study, a formal a priori sample size calculation was not performed, and patients were consecutively enrolled as a convenience sample for exploratory purposes. This approach may have resulted in limited statistical power and increased vulnerability to sample loss, potentially affecting the generalizability of the findings. In addition, the adoption of complete-case analyses represents a methodological limitation, and the findings should therefore be interpreted with caution. Finally, the open-label design inherently introduces potential sources of bias, thereby restricting the internal validity and broader applicability of the conclusions.

## 4. Materials and Methods

### 4.1. Participants and Recruitment Centers

This prospective, multicentric, real-world study enrolled 28 patients (mean age: 31.4 ± 13.4 years) diagnosed with schizophrenia spectrum disorder (DSM-5) and comorbid SUD, consecutively recruited from specialized mental health institutions across Italy. The coordination center was the Hospital Psychiatric Diagnostic and Treatment Service of the University Hospital S.S. Annunziata in Chieti, supported by the Inpatient Psychiatric Center of Villa Maria Pia in Rome, the Day Hospital of Psychiatry and Drug Dependence at the University General Hospital ‘A. Gemelli’ in Rome, and the Psychiatry Outpatient Clinics of the University Hospital ‘San Luigi Gonzaga’ in Turin.

The Psychiatric Diagnostic and Treatment Service of the University Hospital S.S. Annunziata in Chieti is a specialized hospital-based unit dedicated to the evaluation and management of patients presenting with acute psychiatric episodes. The service provides comprehensive diagnostic assessments and individualized treatment planning, delivered by a multidisciplinary team comprising psychiatrists, psychologists, psychiatric nurses, and other mental health professionals. Clinical management includes pharmacological treatment, detailed psychiatric evaluations, and continuous clinical monitoring.

The Inpatient Psychiatric Center of Villa Maria Pia in Rome is a specialized facility for post-acute psychiatric care, offering inpatient treatment for an initial period of 30 days, extendable up to a maximum of 60 days. This setting is designed for patients requiring a higher level of care following an acute psychiatric episode, including individuals discharged from hospital-based psychiatric units or patients whose clinical conditions do not warrant acute hospitalization but still necessitate inpatient treatment. The center focuses on close medication monitoring and the development of medium- to long-term therapeutic programs.

The Day Hospital of Psychiatry and Drug Dependence at the University General Hospital “A. Gemelli” in Rome and the Psychiatry Outpatient Clinics at the University Hospital “San Luigi Gonzaga” in Turin provide specialized psychiatric and addiction services for patients who do not require continuous inpatient monitoring. These services are delivered by multidisciplinary teams including psychiatrists, psychologists, and nursing staff. Clinical activities encompass comprehensive psychiatric evaluations, diagnostic assessments based on clinical interviews and medical history review, the use of standardized assessment tools, and the prescription and monitoring of psychopharmacological treatments, with ongoing evaluation of treatment response and potential adverse effects.

Participants received detailed information about the characteristics of the study drug (quetiapine), the prescribed dosage schedule, and possible side effects before providing written informed consent. Patients were eligible if they were between 18 and 65 years old, diagnosed with schizophrenia spectrum disorder, and had concurrent SUD. Patients with ECG abnormalities (e.g., QTc > 450 ms) were excluded due to the potential cardiac effects of antipsychotic treatment. Individuals with SUD in remission for more than three months were not included to specifically investigate the effects of quetiapine on active substance use and craving. Acute alcohol or substance intoxication was excluded to avoid confounding baseline psychometric assessments. Patients presenting with severe suicidal ideation were excluded because such conditions require urgent and intensive clinical management incompatible with the study protocol. Significant cognitive impairment was considered an exclusion criterion to ensure the capacity to provide informed consent and to complete psychometric evaluations reliably. Finally, pregnant or lactating individuals were excluded in accordance with standard safety and ethical considerations related to psychopharmacological treatment.

### 4.2. Study Design and Treatment Protocol

Patients were categorized according to their prior antipsychotic treatment status. Individuals who were antipsychotic-naive or who had discontinued antipsychotic treatment for at least two weeks were initiated on quetiapine, starting at 25 mg/day and gradually titrated up to a maximum of 400 mg/day according to clinical response. For participants already receiving other antipsychotic medications, the switch to quetiapine was performed due to inadequate clinical response to the prior treatment, and a cross-tapering strategy was implemented to ensure a safe transition. In all cases, dosage adjustments and the final therapeutic dose for each patient were determined by experienced psychiatrists, based on careful clinical assessment of symptom severity, tolerability, and overall clinical response. Baseline anamnestic data and psychometric evaluations were collected at T0, with follow-up assessments at one month (T1) and three months (T2) after treatment initiation. Psychiatric symptoms were measured using the Positive and Negative Syndrome Scale (PANSS) [49]. Substance craving was quantified using a 10 cm Visual Analogue Scale (VAS) [50]. Quality of life was assessed via the Quality of Life, Enjoyment and Satisfaction Questionnaire (Q-LES-Q-SF) [51]. Aggressiveness related to alcohol and substance use was evaluated using the Modified Overt Aggression Scale (MOAS) [52].

Adverse events were systematically documented by an experienced psychiatrist. Moreover, metabolic parameters, including changes in weight and glycemic and lipid profiles, were monitored during follow-up visits through systematic clinical assessment and explicit inquiries directed to both patients and their cohabiting family members who accompanied them.

### 4.3. Statistical Analysis

Statistical analyses were conducted using SPSS (v25) and JASP for Mac (v0.16.4). Descriptive statistics are presented as mean ± standard deviation (SD) for continuous variables and as counts (percentages) for categorical variables. Outcomes were assessed at baseline (T0), 1 month (T1), and 3 months (T2) on the following scales: PANSS Positive, PANSS Negative, PANSS General Psychopathology, MOAS (aggressiveness), VAS Craving, and Q-LES-Q-SF (quality of life). Analyses were conducted on a complete-case basis for each endpoint. No imputation was applied. For each outcome, a one-way repeated-measures ANOVA with Time (T0, T1, T2) as the within-subject factor was used to test change across visits. Assumption of sphericity was evaluated with Mauchly’s test; where violated, Greenhouse–Geisser corrections were applied to the degrees of freedom and corresponding *p*-values. Effect sizes for omnibus tests are expressed as partial eta squared (η^2^p). All tests were two-sided with α = 0.05.

### 4.4. Ethical Considerations

All participants provided written informed consent after being thoroughly informed about the study objectives, treatment regimen, and potential side effects. In cases of legal guardianship, consent was obtained in the presence of a guardian. The study received approval from local ethics committees (protocol n. 7/9 April 2015), institutional review boards, and national regulatory authorities. It adhered to Good Clinical Practice guidelines and the Declaration of Helsinki (1964) and its subsequent amendments [53].

## 5. Conclusions

This study offers preliminary evidence supporting the efficacy of quetiapine in the management of individuals with schizophrenia spectrum disorders, comorbid with substance use disorders.

The results demonstrated meaningful reductions in psychopathological burden, substance cravings, and aggressiveness, alongside notable improvements in quality of life after three months of treatment. Nevertheless, the high rate of non-adherence observed highlights the ongoing challenges in treating this population and underscores the need for individualized therapeutic approaches within integrated mental health and addiction care frameworks.

While these findings suggest a promising role for quetiapine in this complex clinical setting, larger-scale studies with longer follow-up durations are warranted to validate these outcomes and further optimize treatment strategies.

## Figures and Tables

**Table 1:** Demographics and clinical data of the sample (*n* = 28).

Sex, M	22 (78.6%)
Age, years	31.4 ± 13.4 (17–62)
**Substance**	
Cannabis	12 (42.9%)
Cocaine	5 (17.9%)
Alcohol	3 (10.7%)
Polysubstance users	8 (28.5%)
**Diagnosis**	
Substance-induced psychosis	15 (53.6%)
Schizoaffective disorder	7 (25.0%)
Unspecified psychosis	6 (21.4%)
**Concomitant medications at baseline**	
Mood stabilizers	10 (35.7%)
Antipsychotics	6 (21.4%)
Antidepressants	9 (32.1%)
Anxiolytics	12 (42.9%)
Substitution therapy	3 (10.7%)
Other	2 (7.1%)

Abbreviations: M: male.

**Table 2:** Changes in psychometric scales from baseline (T0) to three-month follow-up (T2).

Outcome	Baseline (T0) *n* = 28	One Month (T1) *n* = 16	Three Months (T2) *n* = 16	F	*p*-Value
*PANSS Positive*	15.4 ± 6.9	11.3 ± 3.1	8.7 ± 2.9	12.846	**<0.001**
*PANSS Negative*	16.2 ± 7.0	12.77± 4.5	9.8 ± 3.5	7.933	**<0.001**
*PANSS General psychopathology*	40.5 ± 13.3	32.7 ± 11.18	24.6 ± 7.7	10.560	**<0.001**
*MOAS*	6.9 ± 6.7	4.0 ± 5.5	1.8 ± 3.4	6.117	**0.007**
*VAS Craving*	4.9 ± 2.3	2.69 ± 1.7	1.6 ± 2.0	8.430	**0.002**
*Q-LES-Q-SF*	39.6 ± 9.4	45.9 ± 9.77	51.5 ± 11.1	16.152	**<0.001**

*Abbreviations:* PANSS: Positive and Negative Syndrome Scale; MOAS: Modified Overt Aggression Scale; Q-LES-Q-SF: Quality of Life, Enjoyment and Satisfaction Questionnaire; VAS: Visual analogue scale.

## Data Availability

The original contributions presented in this study are included in the article. Further inquiries can be directed to the corresponding author.
